# Comparative evaluation of efficacy of *Trayodashang Guggul* versus *Rasona Pinda* as an adjuvant with *Katibasti* in the management of *Grudhrasi* (Sciatica):  A randomized controlled trial

**DOI:** 10.12688/f1000research.139568.3

**Published:** 2024-08-12

**Authors:** Mayank Rai, Sadhana Misar, Reeya Gamne

**Affiliations:** 1 Datta Meghe Institute of Higher Education & Research, Wardha, Maharashtra, India

**Keywords:** Grudhrasi, Sciatica, katipradesh, Ruka, Rasonapinda, trayodashang guggul, katibasti, dashmool taila.

## Abstract

**Background:**

In ayurveda, sciatica can be correlated to ‘ *Grudhrasi’* under *Vata Nanatmaja-Vyadhi* (neurological disorders caused by *Vata*, one of the bodily humour). In this mainly bodily humours *vata* and *kapha* are vitiating producing symptoms like piercing pain, stiffness, twitching, numbness and pain radiating from lumbosacral region to lower limb up to the foot. Therapeutic plan includes stabilizing and bringing back the vitiated vata and kapha humours to equilibrium. The prevalence of sciatica varies considerably ranging from 3.8% in the working population to 7.9% in the nonworking population.

**Aim:**

Comparative evaluation of efficacy of *Rasonapinda* and *Trayodashang guggul* as an adjuvant to *katibasti* (oil pooling therapy) in the management of *Grudhrasi* (Sciatica).

**Objectives:**

To assess the efficacy of *Rasonapinda* as an adjuvant to *katibasti* in subjective and objective parameters of *Grudhrasi* (Sciatica). To assess the efficacy of *Trayodashang guggul* as an adjuvant to *katibasti* in subjective and objective parameters of *Grudhrasi* (Sciatica). To compare the efficacy of *Rasonapinda* and *Trayodashang guggul* as an adjuvant to *katibasti* in subjective and objective parameters. Standardisation of *Rasonapinda* (modified formvati).

**Methods:**

In this study, a total of 60 patients will be enrolled and divided equally into two groups. In group A, *Trayodashang Guggul* 500 mg twice a day after meal with warm water for 30 days adjuvant with katibasti for the initial 7 days will be given. In group B, *Rasonapinda* 500 mg twice a day after meal with warm water adjuvant with katibasti for the initial 7 days will be given for 30 days.

**Result:**

The result will be assessed on baseline of subjective and objective parameters and data will be compared after treatment.

**Conclusions:**

It will be based on observations and results obtained.

**Trial registration:**

CTRI No. - CTRI/2022/12/048534 Dated – 27/12/2022.

## Introduction

*Grudhrasi* is a pain dominant ( *Vata Nanatmaja Vyadhi*) neurological disorder. It is identified by pain in the lower back region which radiates downwards to the lumbar, lower back *,* thigh *,* popliteal area *,* calf region *,* and foot. ^1^ *Acharya Charak* has described two types of *Grudhrasi Vata* dominant and *Vata-Kapha* dominant. The *Vata* dominant type of *Grudhrasi* is characterized by pain starting from buttock and then radiates to the lumbar *,* lower back, thigh, popliteal area, calf, and foot along with stiffness, *pain,* pricking sensation, and twitching. Whereas in *Vata-Kapha* dominant type of *Grudhrasi*, aversion to food, drowsiness, and feeling of heaviness are found, which causes the restricted movement of lifting of the leg. ^2^ In modern science *grudhrasi* can be correlated with Sciatica due to similarity in symptoms like severe radiating pain that spreads down one or both legs along the sciatic nerve that originates in the lower back. Sciatica is a crippling condition in which the patient feels pain and/or paraesthesias in the sciatic nerve’s or a related lumbo sacral nerve root’s distribution. ^3^ The disorder has a significant socio economic impact and has the potential to become chronic and irreversible. ^4^ Iin India, 2-40 percent of individuals suffer from sciatica. This occurrence is age-related and unlikely before the age of 20. According to reports, 1 to 10% of the population can suffer sciatica, with individuals aged 25 to 45 being the most commonly affected. ^5^ In modern management of sciatica, mostly non-steroidal anti inflammatory drugs (NSAIDSre used and symptomatic treatment is done. Ayurveda has many more effective methods for treating this excruciating condition. In this disease, mainly Apana and Vyana Vayu (types of vata humour in body) vitiation are observed, but most of the times Kapha humour remain associated in pathogenesis of disease. Therefore, the recommended medicine for treating *Grudhrasi* should be *vata* pacifying *, kapha* pacifying *,* and one which normalizes *vata* movement in body. ^6^ Besides this, medicine should be digestive-carminative, and pain alleviating in properties. In Bhaishajya Ratnavali (one of the classics if ayurveda), *Rasonapinda* is mentioned in the management of *Grudhrasi* having *Rasona* ( *Allium sativum*) as a chief ingredient. ^7^ In phytochemical study, the presence of alkaloids in *Allium sativum* extract shows the potential to have an analgesic, anti-inflammatory, antioxidant and adaptogenic effect. In addition, *Katibasti* (oil pooling therapy) is a successful method for rejuvenation of body tissues that involves applying and retaining specially prepared heated herbal oil inside a border made of flour that is kept in the lower back for a predetermined amount of time. ^8^ It lubricates the joints, strengthens the muscles and connective tissue, and increases flexibility.

Modern therapy includes the use of analgesics, epidural steroid injections and surgery. It has limitations due to side effects and is expensive. In Ayurveda Oleation *,* sudation (steam therapy) *,* blood letting *,* therapeutic heat burn (Agnikarma) *,* enema therapy and palliative therapies are indicated. Palliative therapies are non invasive, simple, safe and cost effective. In classics, various formulations are mentioned for non-invasive therapy like *trayodashang guggul, vatari guggul* etc. ^9^ In Bhaishajya Ratnavali, *Rasonapinda* has been mentioned in the management of *Grudhrasi.* Though *vata* and *kapha* are the bodily humour vitiated in *Grudhrasi*, the impact of digestive fire is not ignored in the pathogenesis of *Grudhrasi.* *Rasonapinda* is totally herbal in nature having analgesic, anti-inflammatory, digestive, carminative properties which will help in correcting weak digestive fire and reducing symptoms like pain, and stiffness. ^10^ Consequently, this research is being done to assess the efficacy of *Rasonapinda* in the management of *Grudhrasi* (sciatica).

### Aim

Comparative evaluation of efficacy of *Rasonapinda* and *Trayodashang guggul* as an adjuvant to *katibasti* (oil pooling therapy) in the management of *Grudhrasi* (sciatica).

### Objectives

1) To assess the efficacy of *Rasonapinda* as an adjuvant to *katibasti* in subjective and objective parameters of *Grudhrasi* (sciatica).
2) To assess the efficacy of *Trayodashang guggul* as an adjuvant to *katibasti* in subjective and objective parameters of *Grudhrasi* (sciatica).
3) To compare the efficacy of *Rasonapinda* and *Trayodashang guggul* as an adjuvant to *katibasti* in subjective and objective parameters.
4) Standardisation of *Rasonapinda* (modified form-vati).

## Methods

### Study setting

The patients will be selected from OPD and IPD of department of kayachikitsa of the Mahatma Gandhi Ayurved College, Hospital & Research Centre, Salod (H) and peripheral camps, mainly the population of Wardha district including patients of all community, (Maharashtra) India. A total of 60 patients will be recruited for the study. They will randomly be divided into two groups; Group A (controlled group) – *Trayodashang Guggul*, and Group B (interventional group) – *Rasona Pinda*. All the baseline parameters will be recorded at the start of the study. The patients will undergo treatment for 30 days for both groups. All the parameters will be recorded at the 0 ^th^, 15 ^th^, 30 ^th^, 45 ^th^ day of the study duration.

**Sample size:** 60 (including sample size calculation).

**Grouping:** Group A (controlled group) – (n=30) *Trayodashang Guggul* as an adjuvant to *katibasti* (oil pooling therapy).

Group B (Interventional group) – (n=30) *Rasonapinda* as an adjuvant to *katibasti* (oil pooling therapy).

All the baseline parameters will be recorded at the beginning of the study. The patients will undergo treatment for 30 days in both groups. Follow ups will be taken on the 15 ^th^ and 30 ^th^ days during treatment and the 45 ^th^ day after treatment.

**Guidelines:** The SPIRIT guidelines were used for the study protocol. ^11^

**Case definition:** Patients between 20–60 years of either sex having classical signs and symptoms of *Grudhrasi* (sciatica) willing to give written informed consent.

**Sampling procedure:** Simple random sampling by computerized table method.

**Type of study:** Interventional study.

**Study design:** Randomized standard controlled single-blind superiority clinical trial.

Simple randomization method will be used to allocate participants in controlled and interventional group. A Random number generator will be used and unique number is given to each participant. Participant with even number will be allocated in controlled group and participant with odd number will be allocated in Interventional group.

**Data monitoring:** formal committee.

### Ethical considerations

The ethical clearance for the publication of this protocol was taken from the I.E.C. committee at Mahatma Gandhi Ayurveda College, Hospital and Research Centre Salod (H), Wardha, Datta Meghe Institute of Medical Sciences with Ref No.-MGACHRC/IEC/july-2022/549 (11/08/22).

CTRI registration for this study – CTRI/2022/12/048534 (27/12/2022).

The committee will decide on the endpoint and oversee the trial as it progresses.

The researcher will assess any adverse event and these will be reported to the ethics committee.

**Consent –** The recruitment of the patient in study groups will be done with written informed consent in their local language while explaining every aspect of the study by the researcher.

Before, during, and after the experiment, the participants’ personal data will be gathered and kept private. The researcher will be the only one having access to the physical data storage facility. Computerised information will be kept on a hard drive that is password-protected and only the researcher can access it.

**Inclusion criteria –** Patients willing to participate with written informed consent.

Patients aged between 20–60 yrs.

Patients having classical sign and symptoms of *Grudhrasi Ruk* (pain), *Stambha* (stiffness), *Toda* (pricking pain), *Muhuspandana* (numbness), *Aruchi* (anorexia), *Tandra* (drowsiness).

Patient fulfilling diagnostic criteria like positive SLRT, Schober’s test, Bowstring Test.

**Exclusion criteria –** Cases of traumatic injury.

Known cases of bone tumour, cancer of spine, tuberculosis of vertebral column, fibrosis of sacral ligaments, and protruded inter vertebral disc except lumbar spondylosis.

Patients with serious health issues such as diabetes mellitus, heart disease, kidney disease, cancer, tuberculosis, and other systemic disorders.

Pregnant and lactating women.

### Withdrawal criteria

If any unintended adverse effects or complications develop while receiving therapy, the patient will be removed from study and receive free care for those issues until they are resolved.

### Intervention description

Group B (Interventional group) – *Rasonapinda* ^12^ 500 mg twice a day with water

Ingredients of *Rasonapinda* are *Allium sativum (Rasona 72 gm), Ferula asafetida (Hingu 12 gm), Cuminum cyminum (Jeeraka 12 gm), Sodiichloridum (Saindhavlavana and Souvarchalalavana 12 gm each), zingiber officinale (Shunthi 12 gm), Piper nigrum (Maricha 12 gm), Piper longum (Pippali 12gm), Ricinus communis (Eranda- Quantity sufficient).*

**Group A** (Controlled group) – *Trayodashang guggul* ^13^^,^^14^ 500 mg twice a day with water

Ingredients of *Trayodashang guggul* are 13 parts *Commiphoramukul (Guggul),* 1 part of each *-Acasia Arabica (Aabha), Withaniasomnifera (Ashwagandha), Juniperus communis (Harpusha), Tinospora cordifolia (Guduchi), Asparagus recemosus (Shatavari), Tribulus terresteris (Gokshur), Argyria speciosa (Vriddhadaru), Pluchea lanceolata (Rasna), Foeniculumvalgare (Shatapushpa), Curcuma zedoaria (Karchur), Trachhyspermumammi (Yavani), Zingiber officinale (Shunthi),* 1/2 part of *Clarified butter (Ghrut).*

**Adjuvent Therapy** *– katibasti* (oil pooling therapy) with *Dashmoola* oil

***Dashmoola oil*** literally means oil prepared of 10 roots of medicinal herb is a polyherbal preparation pacifying all three bodily humours ( *vata, pitta* and *kapha*), more specifically having *vata* pacifying effect mentioned in bhaishajyaratnavali under the management of brain disorders ( *Shirorogadhikar*). It is a well-known for alleviating *Vata* type of bodily humour by oleation therapy. ^15^

Ingredients of *Dashmool* oil are 1 part each of roots of *Aegle marmelos (Bilwa), Premna serratifolia (Agnimantha), Oroxylum indicum (Shyonak), Stereospermum suaveolance (Patala), Gmelina arborea (Gambhari), Desmodiumgangeticum (Shalaparni), Uraria picta (*Prishniparni), *Solanum indicum (Brihati), Solanum surattense (Kantakari), Tribulus terrestris (Gokshura), Vitex negundo (Nirgundi), Brassica comprestris (Sarshap).*

### Drug preparation

*Rasonapinda* will be prepared according to the standard operating procedures outlined in *Sharangdhar Samhita- Madhyam Khand* (ayurveda classics).

*Rasonapinda* will be prepared according to modified form by giving three bhavanas (triturations) of erandmool decoction and converting it into tablet form for easy acceptability and convenience to the patient and further standardization of *Rasona Pinda* will be done. ^16^

### Procedure of Rasona Pinda preparation

Fine powder of all ingredients- *Allium sativum (Rasona 72 gm), Ferula asafetida (Hingu 12 gm), Cuminum cyminum (Jeeraka 12 gm), Sodiichloridum (Saindhavlavana and Souvarchalalavana 12 gm each), zingiber officinale (Shunthi 12 gm), Piper nigrum (Maricha 12 gm), Piper longum (Pippali 12 gm)* will be taken and mixed thoroughly. To this powder, ricinus communis root (erandmool) decoction will be added and triturated for three hours. Such three triturations ( *bhavanas*) will be given. After drying granules will be prepared and tablet of each 250 mg will be prepared by tablet punching machine.

*Trayodashang Guggul* and Dashmool Taila will be procured from a pharmaceutical company.

### Intervention modification

Any unfavourable effects during the course of the treatment will be observed and will be reported to the ethical committee. The patients’ unpleasant effects will be taken care of. Any withdrawal requests from participants will be addressed along with the justification for ending the course of therapy.

### Outcomes

To compare efficacy of *Rasonapinda* and *Trayodashang guggul* as an adjuvant to *katibasti* (oil pooling therapy) in subjective and objective parameters.

### Assessment criteria

Assessment will be done on 0 ^th^, 15 ^th^, 30 ^th^ day during treatment and 45 ^th^ day after treatment

### Subjective criteria – Gradation ^17^

*Ruk* (pain)

*Stambha* (stiffness)

*Toda* (pricking pain)

*Muhuspandana* (numbness)

*Aruchi* (anorexia)

*Tandra* (drowsiness)

Functional disability – Oswestry Disability Assessment Questionnaire

### Objective criteria – (before and after)

SLRT (Straight Leg Raising test)

Walking time – to cover 25 meters

Schober’s Test

Bowstring test

### Oswestry Low Back Pain Disability Questionnaire ^18^

Questionnaire description: Each of the 10 sections that describe the pain and its effects is evaluated from 0 to 5, with higher values indicating a more severe impact.

*Schober’s test*

Locate L5 spinous process (level of posterior superior illiac spine, dimpuls of venus).

Mark 5 cm below and 10 cm above.

Requesting the patient to touch their toes.

Measure the space between the marks.

Normal > 5 cm, Abnormal< 2.5 cm

*Bowstring test*

Subject is supine.

Passively perform SLR on the involved side, if the pain is experienced, flex the subject’s knee to 20 degrees in attempt to reduce the pain.

After that, exert pressure there in an effort to simulate radicular pain.

(+) Tension on the sciatic nerve is indicated by painful radicular reproduction after popliteal compression.

### Participant timeline

**Figure 1.**
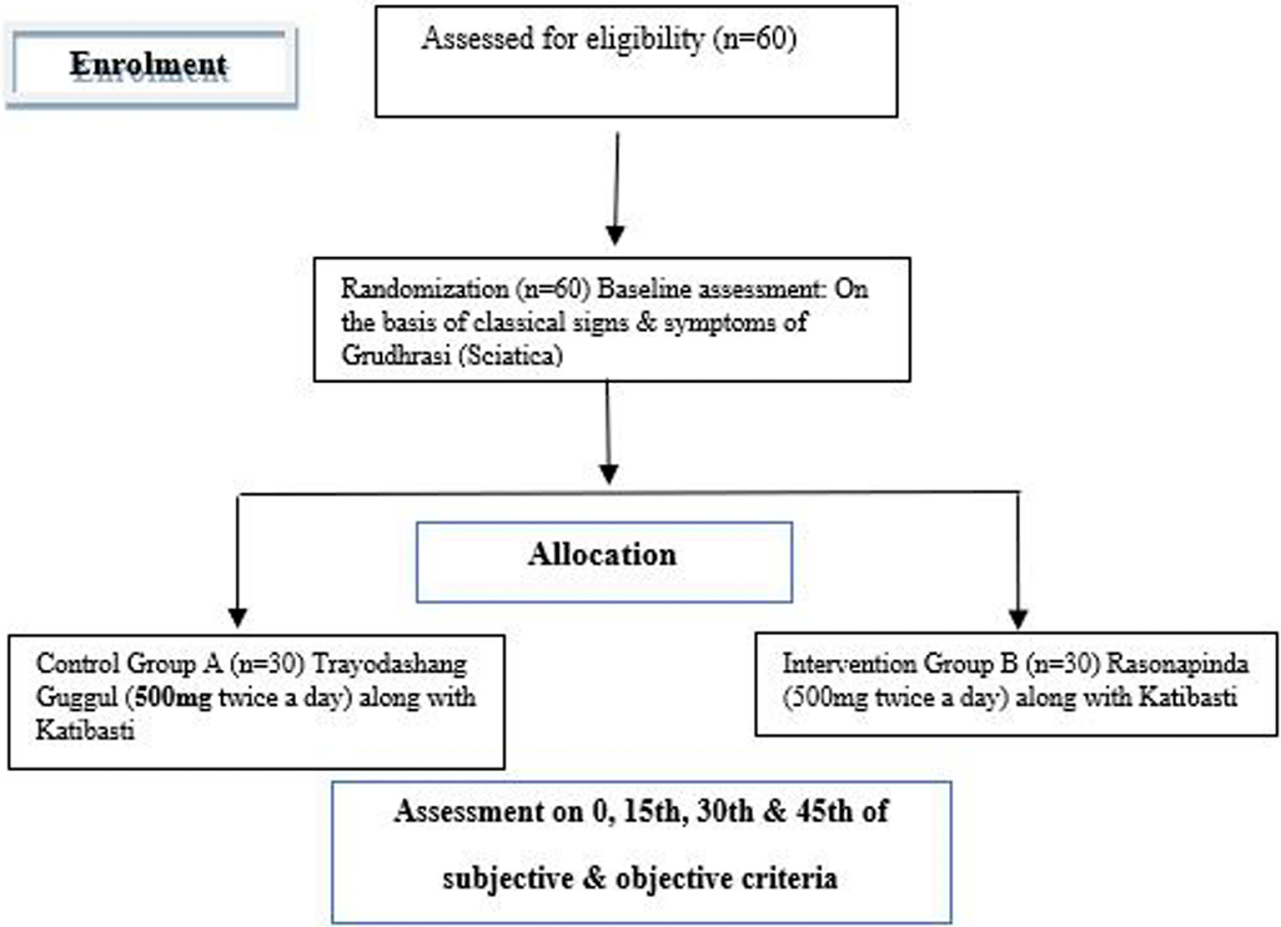
CONSORT flow diagram showing study timeline.

Patients will be treated for 30 days and assessment will be done up to 45 days as mentioned in Figure 1.

### Recruitment

As per sample size calculation, total patients will be recruited. Data collected will be analyzed by using appropriate statistical methods. The patients of *Grudhrasi* will be selected from the Kayachikitsa Out-patient department and In-patient department of Mahatma Gandhi Ayurved College, Hospital & Research Centre, Salod (H), and from specialized peripheral camps.

***Enrolment and interventions time schedule***

The intervention period will be from 0 to 30 days and follow up on the 0 ^th^, 15 ^th^, 30 ^th^ and 45 ^th^ day.

***Sample size***

Formula using Mean difference:

$$n1=n2=2\frac{{\left(Z_{\alpha }+Z_{\beta }\right)}^{2}\sigma^{2}}{{\left(\delta \right)}^{2}}$$

$Z_{\alpha }=1.96$

α=Type I error at 5% at both sides two tailed

$Z_{\beta }=0.84=\text{Power at 80\%}$

Primary Variable = Radiation of Pain

(Mean) value of Radiation of Pain for Trayodashangguggul treatment group (Before) = 2.27 (As per reference article). ^19^

(Mean) value of Radiation of Pain for Trayodashangguggul treatment group (After) = 0.93.

Mean Difference for the effect in Trayodashangguggul after and before 2.27 – 0.93 = 1.34.

Standard Deviation = 0.62

Considering 20% clinically relevant margin for RasonaPinda $\left(\delta \right)$ = (1.34 *35) /100 = 0.469.

Sample size N = $n1=n2=2\frac{{\left(1.96+0.84\right)}^{2}{\left(0.62\right)}^{2}}{{\left(0.469\;\right)}^{2}}=28\;\text{per group}$

Considering 10% drop out = 2

Total sample size required = 28 + 2 = 30 per group.

A total of 60 patients will be recruited for the study.

**Allocation sequence generation** – Computer-Generated Randomization.

**Concealment of allocation** – assessor blinding.

**Allocation implementation** – the first author will generate an allocation sequence, enrol participants, and assign participants to intervention.

**Blinding –** randomized single blind standard controlled superiority clinical trial.

The outcome assessor will be blinded.

Unblinding will not be permissible to outcome assessor.

Participant will be revealed about allocation of intervention at the time of enrolment.

### Data collection plan

Data will be record and assessed as per the subjective and objective criteria on 0 ^th^, 15 ^th^, 30 ^th^ & 45 ^th^ of study (see Table 1).

**Table 1.** The plan for collection of data.

Group	Sample size	Intervention	Dose and frequency	Anupan	Duration	Follow Up
A	30	*Trayodashang Guggul*	500 mg Twice a day after meal	Warm Water	30 days	0 ^th^,15 ^th^, 30 ^th^ day during treatment 45 ^th^ day after treatment
B	30	*RasonaPinda*	500 mg twice a day after meal	Warm water	30 days	
	***Katibasti* (oil pooling therapy) with *Dashmool oil* in both groups**				Initial 7 days	

Ayurveda samhitas

Modern texts

Online search – Google scholar, etc.

60 patients

*TrayodashangGuggul*

RasonaPinda

Case record form

Patients information form

Written and informed consent form

Measuring scale

Sphyghmomanometer

Weighing machine

Data obtained from the follow up chart and other observations will be used and the results will be drawn on the basis of various charts, graphs, and tables. To determine the relevance of the findings, improvement in patient condition as per assessment criteria, after treatment will be noted as significant.

***Drug collection/authentication***

The department of Dravyaguna and Rasashastra of Mahatma Gandhi Ayurved college hospital and Research Center, Salod, Wardha will validate and identify the raw material before it is used to make the medicine. It will be obtained from a reputable source.

***Statistical methods***

Descriptive statistics will summarize baseline characteristics. Baseline comparisons between groups will be conducted using independent t-tests for continuous variables and Chi-square tests for categorical variables. Primary outcome analysis will involve paired t-tests (or Wilcoxon signed-rank tests) within each group to evaluate changes from baseline, and independent t-tests (or Mann-Whitney U tests) to compare changes between groups. Secondary outcomes will be analyzed using repeated measures ANOVA or mixed-effects models for longitudinal data, and Chi-square tests for categorical outcomes. Regression analyses will adjust for potential confounders.

***Study status***

IEC Clearance and CTRI registration has been done. Recruitment of patient has started as per the inclusion and exclusion criteria.

## Discussion

This study will compare the therapeutic efficacy of *Trayodashang guggul* and *Rasonapinda* adjuvant to *katibasti* (oil pooling therapy) in individuals with *grudhrasi* (sciatica). *Grudhrasi* has been mentioned in classics in ayurveda under *vatavyadhi* (neurological disorders caused by Vata, one of the bodily humour) where there is radiating pain along with stiffness in buttocks (sphik) that gradually encroaches posterior aspect of lumbosacral region radiating to the thigh, popliteal area, calf up to the foot sequentially. In ayurveda, treatment means to disintegrates the pathogenesis (samprapti). ^20^ In *grudhrasi* there is mainly vitiation of *vata* humour along with *kapha* humour. ^21^ Also, there is derangement of digestive strength with vitiation of bones and muscles and ligaments by their improper nourishment which gives rise to the above-mentioned symptoms. The quality of drugs for disintegration of disease pathogenesis should include *vata* and *kapha* pacifying action and which also nourishes the body tissues. ^22^ *Trayodashang Guggul* is a polyherbal formulation mentioned in ayurveda in which 13 herbs, including *guggul*, are combined to make *Trayodashang Guggul*, which is then processed in *ghrut* (Ghee). Specifically, constituents like *Asparagus racemosus (shatavari*) *, Winthania somnifera (Ashvagandha*) and *Tinospora cordifolia (guduchi*) are known as rejuvenators and provides strength to body tissues. ^23^ *Trayodashang guggul* also have proven anti-inflammatory action. Additionally *Ghrut* (Ghee) with its catalytic and synergistic action helps in better penetration and absorption of drug. ^24^ Thus *trayodashng guggul* directly has an impact in disintegration of disease pathogenesis and pacifying *vata* humour. *Rasona Pinda* has been mentioned in *Bhaisajya Ratnavali* (Ayurveda classics) which consists of *Rasona* ( *Allium sativum*) *, Trikatu* (combination of *zingiber officinale*, *Piper nigrum* and *Piper longum*) and *errand* ( *Ricinus communis*) as chief ingredients possessing a mainly pungent taste (katu, one of the six taste in ayurveda) and hot in potency. ^25^ *Allium sativum* is a well-known *Kapha* and *Vata* humour pacifying drug. *Rasona* has rejuvenating action ( *Rasayan*). Being rejuvenating, this medication raise the standard of healthy body tissue production and restored normalcy to the vitiation of bone and tissues. *Trikatu* (combination of *zingiber officinale*, *Piper nigrum* and *Piper longum*) helps in *Ama* toxin (undigested food) digestion and enhances digestive fire with its hot potency. ^26^ *Trikatu* (combination of *zingiber officinale*, *Piper nigrum and Piper longum*) has also been shown to be helpful for musculoskeletal diseases, reducing stiffness and swelling. ^27^ Additionally *Rasona Pinda* tablet given is tritutrated with decoction of roots of *Ricinus communis* ( *erandmula*). *Ricinus communis (erandmula)* being sweet *,* pungent *,* astringent in taste and unctuous in nature with hot potency will help in combating vitiated *vata-kapha* bodily humour. ^28^ *Kati Basti* (oil pooling therapy) with *Dashmoola* oil (combination of the roots of 10 herbal drugs) relieves stress from muscles and bones, allowing the *Srotas* (inner channels of body) to function more efficiently. ^29^ It also aids in improved blood circulation and provides flexibility in body movement. Ingredients of *Dashamula* (combination of the roots of 10 herbal drugs) have *Vata* and *Kapha* humour pacifying properties and also possess anti-inflammatory and analgesic action which provides relief from muscular and skeletal discomfort in the back. ^30^

## Conclusion

The trial drug could be efficacious in treating *grudhrasi* (sciatica) as it is a combination of drugs having anti-inflammatory *,* pain alleviating *,* rejuvenating and digestive fire strengthening properties that could be helpful in disintegration of pathogenesis.

### Scope and implications of the proposed study

If *RasonaPinda* will pacify the symptoms in *Grudhrasi* (sciatica) and is found to be helpful to perform routine activities then clinical evidence with simple, safe, cost-effective treatment can be generated for the management of *Grudhrasi* (sciatica).

### Dissemination

This protocol will be further published as a thesis to disseminate the study for Grudhrasi (sciatica). The study protocol provides a detailed overview of the study design, methodology, data collection procedures, data analysis plan, and ethical considerations. By disseminating this protocol, we hope to advance knowledge in the field and facilitate future research.

### Note

The study demonstrates the effectiveness of “Ayurveda,” an old practice practised in several regions of India. If it is determined that this ancient idea is appropriate, it should be thoroughly assessed and partially implemented.

## Data Availability

No data are associated with this article. **Zenodo:** SPIRIT checklist for ‘Comparative evaluation of efficacy of *Trayodashang Guggul* versus *Rasona Pinda* as an adjuvant with *Katibasti* in the management of *Grudhrasi* (Sciatica): A randomized controlled trial’. https://doi.org/10.5281/zenodo.8126753. ^27^ Data are available under the terms of the Creative Commons Attribution 4.0 International license (CC-BY 4.0).

## References

[1] Acharya JT : *Charaka Samhita of Agnivesha, Chikitasthana, Ch. 28, Ver. 56-57.* 4th ed. Varanasi: Chaukhambha Surbharati Prakashana;1994;619.

[2] Acharya JT : *Sushruta Samhita of Sushruta, Nidansthana, Ch. 1, Ver. 74.* 4th ed. Varanasi: ChaukhambhaPrakashana;1980;268.

[3] Sathavane GV Pandya DH Baghel MS : Effect of Vatari Guggulu in the management of Gridhrasi (sciatica). *Ayu.* 2015;36(1):41–45. 10.4103/0974-8520.169019 26730137 PMC4687237

[4] Golwalla AF Nadkar MY : Golwalla’s medicine for students, Part 14 section 3, Root and plexus syndromes.Pg no593.

[5] Reference Source

[6] Bairwa M Mishra PK Sharma B : “BASIC DESCRIPTION OF GRIDHRASI (SCIATICA) ACCORDING TO AYURVED” A LITERARY REVIEW.

[7] Moharana PK Dr. Patel A Dr. : Synergistic Effect of TrayodashangaGuggulu and Yoga Basti in the Management of Low Back Pain with Special Reference to Gridhrasi. *International Journal of Health Sciences & Research.* December 2018;8(12). Reference Source

[8] Ratnavali B : Gobind Das Sen with Vidhyotinihindi commentary by Ambika dutta Shastri, B.R. Vat-vyadhiadhikar, verse-89-90, Edition 1999, published by Chaukhamba Sanskrit Samsthana Varanasi.

[9] Rohini VK Dr. : ARYA AYURVEDIC CENTRE. Reference Source

[10] Ratnavali B : Gobind Das Sen with Vidhyotinihindi commentary by Ambika dutta Shastri, B.R. Vat-vyadhiadhikar, verse-89-90, Edition 1999, published by Chaukhamba Sanskrit Samsthana Varanasi.

[11] Rai M Dr. Wajpeyi SM Dr. Gamne R Dr. : Protocol On-Comparative evaluation of efficacy of Trayodashang Guggul versus Rasona Pinda as an adjuvant with Katibasti in the management of Grudhrasi (Sciatica): A Randomized Controlled Trial (Version v1). *Zenodo.* 2023. 10.5281/zenodo.8126753 PMC11487230 39429641

[12] Bhaisajyaratnavali –shri govind das virachita, vyakhyaakarkavirajsriambikadutt shastri ayurvedacharya, chaukhambhaprakashana, Varanasi, sanvata 2073,5/1406 shirorogachikitsaprakashan 65/111-112.

[13] Ratnavali B : Gobind Das Sen with Vidhyotinihindi commentary by Ambika dutta Shastri, B.R. Vat-vyadhiadhikar, verse-89-90, Edition 1999, published by Chaukhamba Sanskrit Samsthana Varanasi.

[14] Kaviraj BR Sen GD : Edited and Enlarged by Bhaisagratna Sri Brahma Shankar Mishra with “vidyotini” Hindi Commentary by Shri KavirajAmbikadatta Shastri, published by ChaukambhaPrakashan Varanasi, Reprint year. 2016;65:107–109.

[15] Chauhan World JouRnalOf Pharmaceutical Research, A Clinical Evaluation Of The Role Of ‘Shiva Guggulu’ With ‘Dashmool Tail Kati Basti’ In The Management Of Gridhrasi W.S.R. To Sciatica Vol 9, Issue 14, 2020. Iso 9001:2015 Certified Journal

[16] Bharat rathi.renurathi, IJRAP, Pharmaceutical Standardization Of Bakuchi Vati Modified Dosage Form Of Dhatrayadi Yoga, Int. J. RS. Ayurveda Pharma.jan-feb 2017

[17] Chaudhary P : A Clinical Comparative Study On Management Of Grudhrasi (Sciatica) With RasnaGuggulu And TrayodashangGuggulu. *International Ayurvedic Medical Journal.* 2021;9:343–349. 10.46607/iamj0309022021

[18] Oswestry Low Back Pain Disability Questionnaire Sources: Fairbank Jct&Pynsent, Pb (2000) The Oswestry Disability Index. *Spine.* 25(22):2940–2952. discussion 2952. Davidson M & Keating J (2001) A Comparison Of Five Low Back Disability Questionnaires: Reliability And Responsiveness. *Physical Therapy*2002; 82: 8-24. 10.1097/00007632-200011150-00017 11784274

[19] Chaudhary P : A Clinical Comparative Study On Management Of Gridhrasi (Sciatica) With Rasna Guggulu And Trayodashang Guggulu. *International Ayurvedic Medical Journal.* 2021;9:343–349. 10.46607/iamj0309022021

[20] Sharma PV : *Nidanasthana; Vatavyadhi Nidana Adhyaya. Sushruta, Sushruta Samhita. Chapter 1, Verse 74.* Varanasi, India: Chaukhambha Visvabharati;2005;15.

[21] Agnivesha, Charaka Samhita with Charaka Chandrika Hindi Commentary by Dr. Brahmanand Tripathi, Part 1, Sutrasthana 20/11, 1st Edition (reprint), Varanasi: Chaukhambha Krishnadas Academy. 2009; p.389.

[22] Agnivesha, Charaka Samhita with Charaka Chandrika Hindi Commentary by Dr. Brahmanand Tripathi, Part 1, Sutrasthana 20/11, 1st Edition (reprint), Varanasi: Chaukhambha Krishnadas Academy. 2009; p.389.

[23] Gupta S Dey YN Kannojia P : Analgesic and Anti-inflammatory Activities of TrayodashangGuggulu, an Ayurvedic Formulation. *PhytomedicinePlus.* 2022; Volume2(3):100281. 2667-0313. 10.1016/j.phyplu.2022.100281

[24] Moharana PK Dr. Patel A Dr. : Synergistic Effect of TrayodashangaGuggulu and Yoga Basti in the Management of Low Back Pain with Special Reference to Gridhrasi. *International Journal of Health Sciences & Research.* December 2018;8(12). Reference Source

[25] Ratnavali B : Gobind Das Sen with Vidhyotinihindi commentary by Ambika dutta Shastri, B.R. Vat-vyadhiadhikar, verse-89-90, Edition 1999, published by Chaukhamba Sanskrit Samsthana Varanasi.

[26] Charak CS Vidyotini SK Chaturvedi GN , editors. Varanasi:Chaukhamba Bharati Academy. 2003.

[27] Nighantu B Chunekar KC Pandey GS , editors. Varanasi: Chaukhamba Vidyabhavan;1969. 10.5281/zenodo.8126753

[28] Sushruta : *Sushruta Samhita (NibhandasangrahaCommentary of Dalhanacharya and NyayapanijakaCommentary of Gayadasa).* Trikamji editor. 1 ^st^ ed. Varanasi: Chaukhamba Sanskrit Samsthan, Sutra Sthana;2007; Vol.45.

[29] Pooja BA Bhatted IS Meera K : Bhojani’efficacy Of katibasti And Samanausadhi In The Management Of Grudhrasi (Sciatica) - A Comparative Clinical Studyaryavaidyan. Feb. - Apr. 2015; Vol.Xxviii(3): pp.173–177.

[30] Niteshwar K Vidyanath R : *Sahasrayogam; Taila yoga prakarana.* Varanasi, India: Chaukhambha Sanskrit Series Office;2006; vol.1:158.

