# Supplementary material for: Comparative evaluation of efficacy of Trayodashang Guggul versus Rasona Pinda as an adjuvant with Katibasti in the management of Grudhrasi (Sciatica):  A randomized controlled trial
Source: F1000Res. 2024 Aug 12;13:9. Originally published 2024 Jan 3. [Version 3] doi: 10.12688/f1000research.139568.3 (PMC11487230; doi:10.12688/f1000research.139568.3)
Supplement: Peer review history

# Reviewer report: Reviewer response for version 3

**DOI:** 10.5256/f1000research.170138.r328942

**Published:** 2024-10-17

**Authors:** Maheshwor Bhatta

**Affiliations:** 1 Swami Vivekananda Yoga Anusandhana Samsthana, Bengaluru, Karnataka, India

The researcher is doing well, but there are many gaps in the research design(some are already corrected) and presentation( more need to work). The writing lacks clarity and resembles a thesis or dissertation rather than a paper. Conducting research and presenting it as a paper are distinct processes. While I can see the researcher’s good intentions,

 There are numerous repetitive elements throughout the paper, with some points appearing 6-7 times. The paper should be more concise, with improved readability and flow. Here are some specific points for necessary changes:

**In the Introduction:**

1. There are many grammatical mistakes; please check thoroughly.
2. Verify the citation regarding the claim that 2-40 percent of individuals in India suffer from sciatica.
3. Add a sentence highlighting the necessity of this comparative study—what gap in the current literature or practice is being addressed? This should be placed at the end of the introduction.
4. Some parts of the introduction are repetitive (e.g., mentioning Rasonapinda's role in managing Grudhrasi and its analgesic and anti-inflammatory properties). Please condense this information to avoid redundancy.

**In Aims and Objectives:**

- Present the Aims and Objectives in a concise paragraph format for better readability and flow. Merging the ideas into a cohesive narrative will capture the essence of the study more clearly.

**In Methods:**

 (The designation of "randomized controlled single-blind superiority trial" lacks clarity; it appears more like a comparative study. Please specify who is blinded—patients, researchers, or evaluators., Based on your assessment, confirm whether this is a randomized controlled trial. – I got to know that it has already changed, good)

1. Explain why multiple assessments are necessary and their importance in this study.

The paper contains several unnecessary points, particularly concerning the instruments of data collection and details about the ethical committee and CTRI, which are mentioned in multiple places. Additionally, the discussion of data collection is repeated in several sections.

 To enhance the overall flow of the paper, please streamline these areas. Information about sample size and medication is presented in a fragmented manner, with some points appearing earlier and others later. It would be beneficial to consolidate these details into cohesive sections for clarity.

Is the study design appropriate for the research question?

Partly

Is the rationale for, and objectives of, the study clearly described?

Partly

Are sufficient details of the methods provided to allow replication by others?

Partly

Are the datasets clearly presented in a useable and accessible format?

Partly

Reviewer Expertise:

Ayurveda, Clinical Yoga

I confirm that I have read this submission and believe that I have an appropriate level of expertise to confirm that it is of an acceptable scientific standard, however I have significant reservations, as outlined above.

# Reviewer report: Reviewer response for version 3

**DOI:** 10.5256/f1000research.170138.r313360

**Published:** 2024-08-23

**Authors:** Shrilatha Kamath

**Affiliations:** 1 Sri Dharmasthala Manjunatheshwara College of Ayurveda, Udupi, Karanataka, India

The author has made all the necessary changes except for the title correction, which remains unaddressed. I’m finding it challenging to reiterate this point repeatedly. Therefore, I will step back from further corrections and leave the indexing process to proceed as it is. Thank you for your understanding.

Is the study design appropriate for the research question?

Partly

Is the rationale for, and objectives of, the study clearly described?

Partly

Are sufficient details of the methods provided to allow replication by others?

Partly

Are the datasets clearly presented in a useable and accessible format?

Partly

Reviewer Expertise:

Medicine, Chest, Locomotor, ENT, Skin, Autoimmune

I confirm that I have read this submission and believe that I have an appropriate level of expertise to confirm that it is of an acceptable scientific standard.

# Reviewer report: Reviewer response for version 2

**DOI:** 10.5256/f1000research.167923.r296928

**Published:** 2024-07-29

**Authors:** Shrilatha Kamath

**Affiliations:** 1 Sri Dharmasthala Manjunatheshwara College of Ayurveda, Udupi, Karanataka, India

**These were my earlier Suggestions:**

 1.Review of the article: Comparative evaluation of efficacy of *Trayodashang Guggul* versus *Rasona Pinda* as an adjuvant with *Katibasti* in the management of *Grudhrasi* (Sciatica):  A randomized controlled trial

 Title itself is ambiguous- Comparative and controlled words are totally different. This study seems like a comparative study as there is no controlled drug specified

 2. Allocation method is not mentioned. Type of randomising method into two groups has to be mentioned (such as simple or permuted block randomisation etc.)

 3. In methodology, inclusion criteria- Patients having classical signs and symptoms cannot be mentioned. it has to be specific (2-4). Diagnosis cant rely on presence or absence of all the subjective and objective criteria mentioned.

 4. In exclusion criteria IVDP is mentioned. But to exclude MRI is compulsory. As no investigations done is mentioned in the article it cannot be considered. Author has to specify whether only Lumbar spondylosis is included.

**Observations Present**

1. Title is still not rectified   Comparative evaluation of efficacy of *Trayodashang Guggul* versus *Rasona Pinda* as an adjuvant with *Katibasti* in the management of *Grudhrasi* (Sciatica):  A randomized controlled trial
2. Methods- Not corrected   Study setting

The patients will be selected from Kayachikitsa Out-patient department (OPD) and In-patient department (IPD) of the Mahatma Gandhi Ayurved College, Hospital & Research Centre, Salod (H) and peripheral camps, mainly the population of Wardha district including patients of all community, (Maharashtra) India.  A total of 60 patients will be recruited for the study. They will randomly be divided into two groups; Group A – *Trayodashang Guggul*, and Group B – s *Rasona Pinda.* 

1. Patients having classical sign and symptoms of *Grudhrasi Ruk* (pain), *Stambha* (stiffness), *Toda* (pricking pain), *Muhuspandana* (numbness), *Aruchi* (anorexia), *Tandra* (drowsiness).

Patient fulfilling diagnostic criteria like positive SLRT, Schober’s test, Bowstring Test.

1. **Exclusion criteria –** Cases of traumatic injury.

Known cases of bone tumour, cancer of spine, tuberculosis of vertebral column, fibrosis of sacral ligaments, and protruded inter vertebral disc except lumbar spondylosis

Is the study design appropriate for the research question?

Partly

Is the rationale for, and objectives of, the study clearly described?

Partly

Are sufficient details of the methods provided to allow replication by others?

Partly

Are the datasets clearly presented in a useable and accessible format?

Partly

Reviewer Expertise:

Medicine, Chest, Locomotor, ENT, Skin, Autoimmune

I confirm that I have read this submission and believe that I have an appropriate level of expertise to confirm that it is of an acceptable scientific standard, however I have significant reservations, as outlined above.

# Response

**Published:** 2024-08-07

**Authors:** Mayank Rai

**Affiliations:** Datta Meghe Institute of Higher Education & Research, Wardha, Maharashtra, India

Respected Ma'am,

 I have changed the study setting and mentioned controlled group & interventional group, as suggested by you and also made necessary correction as suggested. University SRC Committee suggested title. I have tried making all the correction suggested by you.

 Please look after the changes and do the needful.

# Reviewer report: Reviewer response for version 1

**DOI:** 10.5256/f1000research.152851.r281463

**Published:** 2024-06-20

**Authors:** Vaibhav Bapat

**Affiliations:** 1 National Institute of Ayurveda Deemed University, Jaipur, Rajasthan, India

The work done by the authors to construct this protocol is admirable. There is a demand for complete language editing. Please reconsider the title because control trials are referenced in the title but no specifics about the control group are available anywhere. There might be better optional keywords. Aushadh Sevan Kala is not mentioned. Details of the Katibasti procedure with SOP must be included in this protocol. The inclusion and exclusion criteria appear insufficient. The patient's sex is not mentioned, however pregnant and breastfeeding women are noted in the exclusion criteria. Because the protocol is for 30 days, it is vital to mention whether the katibasti procedure will be continued or discontinued during menstruation. The discussion is written as the study has already been conducted; rather, it should be in accordance with the protocol and should explore the possibility of positive and negative consequences.

Is the study design appropriate for the research question?

Partly

Is the rationale for, and objectives of, the study clearly described?

Partly

Are sufficient details of the methods provided to allow replication by others?

No

Are the datasets clearly presented in a useable and accessible format?

Yes

Reviewer Expertise:

Ayurveda, Skin diseases, Musculoskeletal disorders

I confirm that I have read this submission and believe that I have an appropriate level of expertise to confirm that it is of an acceptable scientific standard, however I have significant reservations, as outlined above.

# Response

**Published:** 2024-06-21

**Authors:** Mayank Rai

**Affiliations:** Datta Meghe Institute of Higher Education & Research, Wardha, Maharashtra, India

Respected Sir/Ma'am,

 I have made all the corrections suggested to me by you. I have mentioned control and interventional groups. *Katibasti *taken as adjuvant therapy in the study only for initial seven days. *Katibasti* will be continued in menstruating females as per their consent if given priority to disease symptoms.

 Thanking you,

 Yours truly,

 Mayank Rai

 PG Scholar 

 MGACH&RC,

 DMIHER,Wardha

# Reviewer report: Reviewer response for version 1

**DOI:** 10.5256/f1000research.152851.r281465

**Published:** 2024-06-11

**Authors:** Shrilatha Kamath

**Affiliations:** 1 Sri Dharmasthala Manjunatheshwara College of Ayurveda, Udupi, Karanataka, India

Review of the article: Comparative evaluation of efficacy of *Trayodashang Guggul* versus *Rasona Pinda* as an adjuvant with *Katibasti* in the management of *Grudhrasi* (Sciatica):  A randomized controlled trial

 1. Title itself is ambiguous- Comparative and controlled words are totally different. This study seems like a comparative study as there is no controlled drug specified

 2. Allocation method is not mentioned. Type of randomizing method into two groups has to be mentioned (such as simple or permuted block randomization etc.)

 3. In methodology, inclusion criteria- Patients having classical signs and symptoms cannot be mentioned. it has to be specific (2-4). Diagnosis can't rely on presence or absence of all the subjective and objective criteria mentioned.

 4. In exclusion criteria IVDP is mentioned. But to exclude MRI is compulsory. As no investigations done is mentioned in the article it cannot be considered. Author has to specify whether only Lumbar spondylosis is included.

 5. Throughout the study assessment is mentioned on 0,15,45 days. But in the Participant timeline- it is written as:

 Patients will be treated for 60 days and assessment will be done up to 90 days as mentioned in.... which doesn't match at all.

 6. Statistical test: "As the study is randomized controlled trial, both parametric and non parametric tests like independent t-test, paired t-test, and Man-Whitney statistical test will be applied to the data generated":

 a. This study is neither randomized nor controlled

 b. Non Parametric test has to be Wilcoxon signed rank test and Mann Whitney along with t test

Is the study design appropriate for the research question?

Partly

Is the rationale for, and objectives of, the study clearly described?

Partly

Are sufficient details of the methods provided to allow replication by others?

Partly

Are the datasets clearly presented in a useable and accessible format?

Partly

Reviewer Expertise:

MedicineKayachikitsa

I confirm that I have read this submission and believe that I have an appropriate level of expertise to confirm that it is of an acceptable scientific standard, however I have significant reservations, as outlined above.

## References

[1] : Comparative evaluation of efficacy of Trayodashang Guggul versus Rasona Pinda as an adjuvant with Katibasti in the management of Grudhrasi (Sciatica): A randomized controlled trial. *F1000Research* .2024;13: 10.12688/f1000research.139568.1 10.12688/f1000research.139568.1 PMC1148723039429641

# Response

**Published:** 2024-06-13

**Authors:** Mayank Rai

**Affiliations:** Datta Meghe Institute of Higher Education & Research, Wardha, Maharashtra, India

Respected Mam/sir

 I have made all the necessary corrections suggested by you.

 I have specified the sampling procedure,inclusion-exclusion criteria, statistical analysis plan, study duration, and rest of corrections.

 kindly please consider my manuscript for further proceedings.

 thanking you

 your's faithfully

 Mayank Rai

 PG Scholar

 MGACH&RC

 Datta meghe institute of higher education & research center
